# Overnight negative emotion inertia is moderated by evening cortisol levels

**DOI:** 10.1186/s40359-026-05017-z

**Published:** 2026-06-26

**Authors:** Lennart Seizer, Keisuke Takano, Johanna Löchner

**Affiliations:** 1 Institute for General Practice and Family Medicine, LMU University Hospital, Munich, Germany; 2 German Center for Mental Health (DZPG), Munich, Germany; 3 Department of Psychology, Clinical Psychology and Psychological Treatment, Ludwig-Maximilians-Universität München, Munich, Germany; 4 Human Informatics and Interaction Research Institute, The National Institute of Advanced Industrial Science and Technology (AIST), Tsukuba, Japan

**Keywords:** Emotion, Inertia, Cortisol, Sleep

## Abstract

Emotional inertia refers to the stability of emotional states over time and has been linked to overall well-being and mental health. This pilot study examines overnight emotional inertia, that is the carry-over of emotions across the sleep period, from evening to morning, and particularly its relationship with evening cortisol levels. 26 female primary caregivers reported their emotional states and provided saliva samples for cortisol measurement each morning and evening over two weeks. Multi-level models were used to regress morning emotions on evening emotions and cortisol levels. Thereby, evening cortisol levels were found to moderate the overnight inertia of negative emotions, with higher cortisol levels associated with stronger persistence of negative emotions from evening to morning. These findings suggest that elevated evening cortisol levels enhance the carry-over of negative emotions into the next day, potentially linking stress-related physiological processes with overnight emotional inertia. These findings provide preliminary evidence that evening HPA-axis activity may be associated with the overnight persistence of negative affect, although replication in larger and more heterogeneous samples is needed.

## Introduction

Emotional inertia refers to the persistence or stability of emotional states over time. It is quantified by the degree to which an individual’s current emotional state can be predicted by their previous emotional states, typically operationalized through (lag-1) autocorrelations or (first-order) autoregressive models, whereby emotional states at time *t* are predicted by the preceding state (*t*-1) [1]. These models are typically fit over consecutive measurements throughout the day (e.g., using ecological momentary assessment) and have been shown to yield positive estimates over timeframes of seconds, minutes, hours, and days, indicating that emotional states persist to some extent over time [2]. However, the magnitude of this autoregressive effect varies across individuals. Thereby, high emotional inertia suggests a strong continuity of emotions from one moment to the next and implies that the individual’s emotions are less influenced by new events or stimuli with a weaker homeostatic force that pulls emotions back towards baseline following perturbations. High inertia of negative emotions has been linked to poor well-being and mental health and may serve as a transdiagnostic vulnerability factor for psychopathology in the sense of critical slowing down [2–5], although it has been argued that emotion dynamics add little additional predictive value to the mean levels [6, 7]. On the other hand, high inertia of positive emotions, i.e., the capacity to carry over positive affect from one moment to the next, appears to play a role in the prevention and recovery from depressive symptoms [8].

Contrary to the emotional inertia of consecutive states through the day, less studies have investigated overnight emotional inertia, that is, the carry-over of emotions across the sleep period, from evening to morning. Generally, two opposing theories exist: (1) the idea of conservation of emotional momentum posits that emotions are resistant to change over time unless acted upon by a force [9], while, on the other hand, (2) sleep-dependent reorganization processes might aid in the resolution of distressing experiences and to reset emotional states [10, 11]. In a layman survey on people’s implicit theories on the matter, no agreement was found: About half of the participants believed that their evening emotions persisted into the morning following a night’s sleep, while the other half believed that their emotions were reset to a starting point following a night’s sleep [12]. In previous intensive longitudinal studies that regressed morning on evening emotions, Minaeva et al., [13] found significant overnight inertia in depressed and non-depressed women, for both, positive and negative emotions, while Frérart et al., [12] found a significant effect only for negative emotions. Further, like daytime emotional inertia, overnight inertia of negative emotions has been associated with clinical depression and symptom severity, although this relation was only found in clinically depressed individuals but not in healthy subjects [12, 13].

The neurobiological basis of emotional inertia has not been fully understood yet [2]. For overnight inertia, mostly sleep characteristics have been considered as potential mechanisms of emotion continuity or reset. Sleep plays an important role in the modulation of emotional memory formation and maintenance of emotional homeostasis [10, 11, 14]. Further, sleep helps in the processing of emotional experiences, regulation of emotions, and restoration of appropriate next-day emotion reactivity [15]. Sleep characteristics during the night have been shown to influence how we feel on the following day. For example, sleep quality has been associated with higher positive emotions and lower negative emotions, while the opposite was true for sleep loss [16, 17]. Furthermore, an observational study conducted on adolescents investigated the emotional inertia of daily stress-related emotions overnight [18]. There were, on average, only minor and non-significant emotional inertia effects, but in people who slept for shorter periods of time at night, these effects were stronger. Conversely, the emotions experienced throughout a day can impact sleep characteristics. Negative emotions and repetitive thinking in the evening have been linked to decreased sleep quality with increased sleep fragmentation and disturbance [19–21].

Given this limited and mixed evidence, further intensive longitudinal research on overnight emotional inertia is needed. In the present pilot study, participants reported their emotional state each morning and evening over a two-week period, allowing us to examine the carry-over of emotions from evening to the following morning. In addition, salivary cortisol was measured each evening as a potential moderator of this overnight process. Cortisol is the main effector hormone of the hypothalamus-pituitary-adrenal (HPA) axis and follows a pronounced diurnal rhythm, with levels typically peaking around the sleep-wake transition and declining across the day to low evening levels [22]. In this context, evening cortisol can be understood as a naturalistic end-of-day indicator of HPA-axis activity. Relatively elevated evening cortisol may indicate a flatter diurnal cortisol slope or less complete decline across the day, patterns that have been linked to stress-related dysregulation and poorer emotional health [23, 24]. Because momentary negative affect is positively associated with cortisol in daily life, elevated evening cortisol may also be relevant to the persistence of negative emotional states overnight [25]. Prior work has linked perceived stress and diurnal cortisol to daytime emotional inertia [26, 27], anticipated stress to next-day awakening cortisol levels [28], and elevated evening cortisol has also been associated with short sleep duration and sleep disturbances [29, 30]. At the same time, salivary cortisol is sensitive to biological and methodological influences, including sampling time and recent behavior, which should be considered when interpreting ambulatory cortisol values [31]. Against this background, we examined whether evening cortisol moderates overnight emotional inertia. We hypothesized that (1) negative emotional states in the evening would positively predict negative emotional states the following morning, (2) positive emotional states in the evening would positively predict positive emotional states the following morning, and (3) higher evening cortisol would be associated with stronger overnight emotional inertia for both negative and positive emotions.

## Methods

### Study design

In this study, 26 female participants were observed over a span of two weeks. Throughout this period, they filled out a short ambulatory questionnaire on their emotional state each morning and evening. Concurrently, they provided saliva samples via passive drool at the same occasions. All participants were the primary caregiver of an infant. Given the inherently stressful nature of parenting infants, participants were allowed to adjust the sampling times to their needs to enhance protocol adherence and reduce study-related disruption [1]. In the morning the participants were instructed to complete the sampling not earlier than one hour after awakening. Consequently, the average sampling times were 08:47 (*SD* = 83 min) in the morning and 18:28 (*SD* = 49 min) in the evening. All subjects provided written informed consent for their participation and for the publication of data. The Ethics Committee of the University Hospital Tübingen approved this study.

### Participants

The sample consisted of 26 female participants without mental disorders, with an average age of 33.55 years (*SD* = 3.59, *Min* = 28, *Max* = 38). All participants were the primary caregiver of an infant. Their children were 58% male and aged on average 28.6 months (*SD* = 11.3, *Min* = 13, *Max* = 49). The participants worked on average 25.15 h per week (*SD* = 8.58). All participants were born in Germany and identified as German nationals. Participants were mostly recruited from local day-care centers (e.g., flyers and bulk mails) and participation was incentivized with a 50€ voucher .

### Emotion questionnaire

The ambulatory questionnaire was completed on the online platform *SoSci Survey* (Leiner, 2019). Every participant was provided with a unique link that they could access from their own smartphones throughout the study period. The emotion questionnaire comprised seven items, each rated on a five-point Likert scale. Three items were combined to create a positive emotion scale (*happy*, *interested*, and *lively/active*), while four items formed the negative affect scale (*angry*, *anxious*, *sad*, and *tired/exhausted*), by averaging the individual item scores [32]. To provide psychometric information appropriate for intensive longitudinal data, we estimated intraclass correlations (ICCs) and reliable-change coefficients [33]. Positive emotion showed an ICC of 0.06 and a reliable-change coefficient of 0.50. Negative affect showed an ICC of 0.09 and a reliable-change coefficient of 0.56, indicating that the scales captured substantial within-person fluctuation across measurement occasions.

### Cortisol measurement

At each measurement occasion, participants collected samples immediately after completing the ambulatory questionnaire. They were instructed to avoid food and drink, except water, for 30 min before saliva collection and to freeze the samples promptly after collection. After the 2-week period, the samples were transported to the laboratory in a frozen state and stored at -80 °C until analysis. Salivary cortisol levels were measured through enzyme-linked immunosorbent assays (ELISA; TECAN, RE52611) following the manufacturer’s guidelines. Each biochemical analysis was conducted at least twice, with the results averaged across multiple runs. Cortisol concentrations are given in nanograms per milliliter (ng/ml). Cortisol concentrations in saliva have been shown to be stable at room temperature for up to seven days and frozen for up to three months [34–36].

### Data analysis

Multilevel regression models with full random effects and autocorrelated errors were used to accommodate the nested data structure with observations (level 1) being clustered within individuals (level 2). In total, four different models were estimated. Model 1 and 2 only concerned with the overnight inertia of positive and negative emotions, which was operationalized as the effect of the previous day evening emotional state on the current day’s morning emotional state over the 14 days. The model was estimated separately for positive and negative emotions. In Model 3 and 4, the evening cortisol levels and an interaction term between evening cortisol and evening emotions were added to Model 1 and 2 as predictors to test the potential moderation of the overnight emotion inertia effect by cortisol levels. The models can be described as

$$\:Model\:1\:and\:2:$$

$$\:{E\_mor}_{id}={\beta\:}_{0i}+{\beta\:}_{1i}*{E\_eve}_{id-1}+{\epsilon\:}_{id}$$

$$\:Model\:3\:and\:4:$$

$$\begin{aligned}\:{E\_mor}_{id}&={\beta\:}_{0i}+{\beta\:}_{1i}*{E\_eve}_{id-1}\\&+{\beta\:}_{2i}*{Cort}_{id-1}\\&+{\beta\:}_{3i}*{E\_eve}_{id-1}\\&*{Cort}_{id-1}+{\epsilon\:}_{id}\end{aligned}$$

with *E_mor*_*id*_ and *E_eve*_*id*_ representing the emotional state each morning for individual *i* at day *d*, or rather at the previous day (*d* − 1) and *ε* as the level-1 error component. In the moderation models (3 and 4), cortisol levels (*Cort*) are included as an additional predictor. To differentiate within-person effects from between-person effects, we computed person-mean centered variables for all predictors. Model diagnostics were examined to assess assumptions of the multilevel models, including residual normality, homoscedasticity, and the adequacy of the autocorrelation structure. The sample was part of a larger study on parenting stress and parent-child interactions; thus the sample size calculations were performed for the overarching project. Because the moderation models were not powered a priori for the focal interaction term, we conducted a sensitivity analysis. Using a Wald-type approximation based on the observed standard error of the interaction coefficient, the minimum detectable interaction effect at 80% power and α = 0.05 was approximately b = 0.22 for the negative-affect model and b = 0.36 for the positive-affect model. These values should be interpreted as approximate indicators of model sensitivity rather than definitive power estimates, as they were derived from observed standard errors in a relatively small multilevel sample. All statistical analyses were performed in *R* 4.4 [37] using the *nlme* package [38].

## Results

There was high compliance for questionnaire completion (*M* = 92.72%, *SD* = 5.76%) and saliva collection (*M* = 88.31%, *SD* = 7.65%), but four participants dropped out early and did not return any saliva samples. These participants were only included in Models 1 and 2. The average positive emotion score was 3.04 (*SD* = 1.13) and the average negative emotion score was 1.89 (*SD* = 0.80).

The results from the multi-level models predicting morning affect from evening affect are summarized in Table 1. We found no significant effect of evening emotions on morning emotions (Models 1 and 2), meaning that emotional states in the evening did not predict the emotional state the next morning. However, we found a significant interaction effect between evening negative emotions and evening cortisol levels, such that higher cortisol levels were associated with a more positive association between negative emotional states in the evening and the next morning (Models 3 and 4).

**Table 1:** Results of the multi-level models. The estimates are unstandardized regression coefficients, with standard errors (SE), and 95% confidence intervals (CI). Models 1 and 2: *N* = 26 subjects, 288 time points. Models 3 and 4: *N* = 22 subjects, 229 time points

	Negative emotion				Positive emotion			
	b	SE	CI	*p*	b	SE	CI	*p*
Models 1 and 2: Overnight inertia								
Intercept	1.77	0.07	1.64;1.90		3.18	0.08	3.01; 3.34	
Evening emotion	0.03	0.07	-0.10; 0.16	0.616	-0.02	0.07	-0.14; 0.11	0.819
Models 3 and 4: Moderation by cortisol								
Intercept	1.78	0.07	1.64; 1.92		3.24	0.08	3.08; 3.40	
Evening emotion	0.05	0.08	-0.11; 0.20	0.579	-0.03	0.08	-0.18; 0.13	0.741
Evening cortisol	-0.04	0.06	-0.17; 0.09	0.559	0.04	0.09	-0.14; 0.21	0.696
Cortisol x emotion	0.19	0.08	0.03; 0.36	0.018	0.12	0.13	-0.13; 0.36	0.348

Because assessment times varied across days, we conducted sensitivity analyses using the same mixed-model framework as in the main analysis while additionally adjusting for morning assessment time, previous-evening assessment time, and the elapsed time between the previous evening and the next morning. The evening negative affect × evening cortisol interaction remained positive when adjusting for morning and evening assessment time (*b* = 0.56, *SE* = 0.55, *p* = .313), when adjusting for the elapsed interval (*b* = 0.24, *SE* = 0.33, *p* = .463), and when adjusting for all three timing covariates simultaneously (*b* = 0.29, *SE* = 0.34, *p* = .399).

## Discussion

In this study, 26 female participants completed an emotion questionnaire each morning and evening during their everyday life and collected saliva samples for determination of cortisol concentrations at the same timepoints. We found the overnight inertia of negative emotions to be moderated by evening cortisol levels. Thereby, higher cortisol was associated with a stronger overnight inertia effect, relative to each subject’s baseline. The fact that this moderation was only found for negative, but not for positive emotions may be explained by the findings that negative emotions exhibit stronger overnight inertia than positive emotions [12] and that negative emotions are rather associated with stress system activity, such as HPA, than positive emotions. However, we did not find significant overnight inertia effects for positive and negative emotions per se. This might be a feature of our sample, as overnight inertia has been hypothesized to be related to sleep characteristics and the irregular sleep pattern of new parents can deviate from other people. Still, this is an interesting finding, since emotional inertia is mostly studied in mental health science with sleeping problems being a transdiagnostic symptom in many mental disorders [39].

Importantly, the moderation effect was no longer statistically significant in the timing-adjusted sensitivity analyses. Although the interaction remained positive across all adjusted models, these findings suggest that the effect may be sensitive to variability in assessment timing. Consequently, the present results should not be interpreted as robust evidence for a stable moderation effect but rather as an initial signal that warrants replication in larger and more highly standardized studies.

Cortisol levels typically follow a diurnal pattern, peaking in the morning and declining throughout the day [22]. Changes in this pattern like elevated evening levels are associated with disrupted sleep [29, 30] and impaired emotional memory consolidation during sleep [40], which, in turn, may reinforce negative emotional states in the morning [16, 17]. Additionally, negative emotional states during everyday life are linked to increased levels of cortisol [25], providing a potential psychoneuroendocrine pathway of overnight emotional inertia. Moreover, stressful incidents during the day can cause negative emotions [41], increase cortisol levels [42], and lead to poor sleep [43, 44]. Fittingly, stress has also been associated with increased inertia of negative emotional states [27]. This cascade of negative emotions, HPA axis activity, and sleep could explain the found moderation of overnight inertia by evening cortisol levels and may represent mechanisms by which stress influences the continuity of emotional states throughout the night.

There are limitations of this study, like the small sample size, limited to female primary caregivers of infants, that restricts the generalizability of the findings. Additionally, the study did not include measures of sleep characteristics and relied on self-reported emotional states at the level of positive and negative emotions. In comparison to the trajectories of the individual emotions they include, these aggregated scales may exhibit distinct temporal patterns [45, 46]. Further, as the primary aim of the study was not the investigation of overnight inertia, the sampling was not timed to be immediately prior to bedtime and after awakening but occurred in 12 h-intervals. Thus, incidents that happened between the assessment and bedtime in the evening or between awakening and the assessment in the morning could have biased the immediate overnight effect [47]. As sleep variables were not assessed we could not examine how closely the evening samples reflected proximity to sleep. This is particularly relevant for cortisol, given its pronounced diurnal rhythm, and it limits the interpretation of the measurements as precise circadian markers. Accordingly, the cortisol values in the present study should be interpreted as ambulatory morning and evening samples obtained under naturalistic conditions rather than as standardized assessments relative to bedtime.

A strength of this study is the intensive longitudinal design, which allowed for detailed tracking of emotional and physiological changes over two weeks [48]. Thereby, our study contributes to the understanding of overnight emotional inertia and the moderating role of evening cortisol. The findings suggest that higher evening cortisol levels can strengthen the persistence of negative emotions across the sleep period. Taken together, these findings suggest a potential association between evening HPA-axis activity and the overnight persistence of negative affect, although the timing-sensitive nature of the results warrants cautious interpretation. Although sleep-related mechanisms are plausible and discussed in the broader literature, sleep was not directly measured in the present study; thus, any role of sleep in explaining the observed moderation remains speculative. Further, comparison of our findings with earlier research is limited since there only few studies investigated overnight emotional inertia directly. Thus, future research should aim to replicate these findings in larger, more diverse samples and explore additional biomarkers, emotion regulation, and measures of sleep architecture that may influence emotional inertia. Examining the bidirectional relationship between stress system activity, sleep, and emotions could provide deeper insights into how emotional experiences are transferred from day to day, with potential implications for mental health research [49].

## Data Availability

The data are available upon request from the corresponding author.
